# The correct interpretation of thyroid FNAB cytological result by the endocrinologist as a basis for further optimal diagnostics and treatment

**DOI:** 10.1186/1756-6614-6-S2-A38

**Published:** 2013-04-05

**Authors:** Andrzej Lewiński

**Affiliations:** 1 Department of Endocrinology and Metabolic Diseases, Medical University of Lodz, Poland; 2 Polish Mother's Memorial Hospital – Research Institute, Lodz, Poland

## 

Adopted by the Polish Society of Endocrinology and the Polish Thyroid Association American system of the classification of FNAB cytological smears - the Bethesda System for Reporting Thyroid Cytopathology (TBSRTC) determines exactly how to run diagnostics and therapy for each category of smears. Cytopathologist establishing a diagnosis should provide it in such a way as to include a detailed description of both the observed specimen [existing types of cells, their abundance (their number) and the spatial arrangements (layouts) which they form, as well as other components of the smear, such as, e.g. colloid protein masses] and a diagnostic conclusion which assigns a smear to one of six (6) categories TBSRTC. In order to avoid unintentional mistake during the process of assigning in question, the endocrinologists should have a basic idea of what kind cellular and/or acellular components determine and prejudge the qualification of cytological smear to a specific TBSRTC category. It should be noted that having this skill is not to control the description of cytological result as morphological diagnostics (within the meaning of the direct microscopic evaluation of slides) is beyond the reach of an endocrinologist and other doctors who are not cytopathologists, just check whether there is compliance of the diagnostic conclusion and morphological description of the smear, because it is TBSRTC category which decisively determines further medical management. As shown by us in the proposed algorithm, the assignment of the specimen to each category, together with, the so-called, US risk pattern (which is defined in a 3-stage as a pattern of high, intermediate or low risk) are decisive for the recommendation of surgical treatment or abandonment of such treatment and further follow up; they also are decisive as regards the frequency of repeat US and FNAB cytology. Thus, the essence of the present lecture is to familiarize endocrinologists with the significance of individual components of the smear, as described in cytology, in determining the appropriate TBSRTC category. In practice, the most important issue is to properly differentiate categories II, III and IV, because there is little doubt as regards treatment of patients with other smear categories (I, V and VI). Therefore, it is necessary to remember that the normotypic thyroid follicular cells (tfc), both dispersed and arranged in large and/or medium-sized spatial patches and groups, and sometimes nests, are characteristic rather for benign lesions. On the other hand, small spatial layouts, especially arrangements of rosettes type are characteristic for category IV (follicular neoplasm or suspicious for a follicular neoplasm). It should be added, however, that large spatial layouts may also be present in papillary thyroid carcinoma; undoubted help in case of detection of this cancer is, however, characteristic appearance of cells and their typical cytological features. Cytological smears obtained from benign lesions may contain cells with different abundance, and also a variety of other cells can be the smear components, such as, e.g. cells typical for inflammatory processes (lymphocytes, neutrophils, multi- and mononucleated macrophages), anisocytosis is frequently observed (sometimes anisocaryosis), and - which is particularly characteristic - considerable quantities of colloid are present in the smear. In contrast to the above components, "follicular neoplasm" in cytological smear has no colloid at all or its trace amount, monomorphic tfc occurring usually in large numbers and arranged in small layouts, which characteristically take the form of the, so-called, rosettes. Anisocytosis generally does not occur, cell types other than monomorphic tfc and larger spatial arrangements are very rare. In case of smears collected from oncocytic lesions, the assignment to the category IV is determined by the percentage of oncocytes in the smear (above 75%). Oncocytic cells should have described the presence of nucleoli, anisocytosis may occur in this case and spatial arrangements can be of any type. Finally, it is noted that smears of category III, called by some authors as "a category of exclusion", contain both components characteristic for category II and may also include elements of morphological smears of category IV, but in a such proportion that would not allow to categorically qualify them as benign lesions or as “a suspicion of follicular neoplasm”.

